# Gastric Cancer: Mechanisms, Biomarkers, and Therapeutic Approaches

**DOI:** 10.3390/biomedicines10030543

**Published:** 2022-02-24

**Authors:** Sangjoon Choi, Sujin Park, Hyunjin Kim, So Young Kang, Soomin Ahn, Kyoung-Mee Kim

**Affiliations:** 1 Department of Pathology and Translational Genomics, Samsung Medical Center, Sungkyunkwan University School of Medicine, Seoul 06351, Korea; choisj88@gmail.com (S.C.); sujin423.park@samsung.com (S.P.); hkim.pathol@gmail.com (H.K.); sy500.kang@samsung.com (S.Y.K.); soomin17.ahn@samsung.com (S.A.); 2 Center of Companion Diagnostics, Samsung Medical Center, Seoul 06351, Korea

**Keywords:** gastric cancer, precision, biomarker, targeted therapy, immunotherapy

## Abstract

Gastric cancer (GC) remains one of the most common deadly malignancies worldwide. Recently, several targeted therapeutics for treating unresectable or metastatic GC have been developed. Comprehensive characterization of the molecular profile and of the tumor immune microenvironment of GC has allowed researchers to explore promising biomarkers for GC treatment and has enabled a new paradigm in precision-targeted immunotherapy. In this article, we review established and promising new biomarkers relevant in GC, with a focus on their clinical implications, diagnostic methods, and the efficacy of targeted agents.

## 1. Introduction

Gastric cancer (GC) is one of the most common malignant tumors and is the fourth leading cause of cancer-related deaths worldwide [1]. Locally advanced and metastatic GC generally have a poor prognosis despite chemotherapy and remain therapeutic challenges. Thus, both clinicians and patients benefit greatly from the availability of advanced treatment options. The rapid development of cancer biology and sequencing techniques has enabled precise profiling of cancer mutations and the tumor immune microenvironment of GC, which are crucial for therapeutic decisions [2,3].

GC is a heterogeneous disease with diverse histologic and genomic subtypes. Based on genomic and epigenomic alterations, The Cancer Genome Atlas (TCGA) research program proposes four molecular subtypes of GC: Epstein-Barr virus (EBV), microsatellite instability (MSI), genomically stable (GS), and chromosomal instability (CIN) [4]. These four subtypes have distinct clinicopathological and molecular characteristics as well as distinct tumor immune microenvironments (Table 1) [3,4,5]. As a result of an improved understanding of the molecular profiles of GC, new targets and drugs have been discovered, and there have been some remarkable success stories [2,6]. Large clinical studies have demonstrated that molecular-targeted therapies of GC involve diverse mechanisms [6]. In the era of precision cancer therapy, a comprehensive understanding of the underlying targeting mechanisms and of predictive biomarkers with matched therapeutic agents can provide new insights for GC treatment.

To date, only three molecular biomarkers have been demonstrated to predict responses to targeted therapies in GC [2]: human epidermal growth factor receptor 2-positive (HER2+) GC for trastuzumab and for trastuzumab deruxtecan, and microsatellite instability-high (MSI-H) and programmed death-ligand 1-positive (PD-L1+) GC for pembrolizumab. Nevertheless, an increasing number of biomarker-based clinical trials have demonstrated the efficacy of targeted therapy alone or in combination with conventional chemotherapies [2,6]. Considering the low frequency of targets (biomarkers) in GC, the accurate detection of target molecules by adequate diagnostic methods is crucial for patient selection for targeted therapy. 

In this review, we describe the key biomarkers in GC along with their clinical significance, detection methods, limitations, associated predictive markers, and targeted agents. In particular, we focused on well-established (PD-L1, HER2, and vascular endothelial growth factor receptor 2 (VEGFR2)) and emerging (mesenchymal-epithelial transition (MET), fibroblast growth factor receptor 2 (FGFR2), Claudin 18.2 (CLDN18.2), and tumor-infiltrating lymphocytes (TIL)) biomarkers (Table 2).

## 2. Currently Established and Emerging Biomarkers in GC

### 2.1. PD-L1

**Figure 1:**
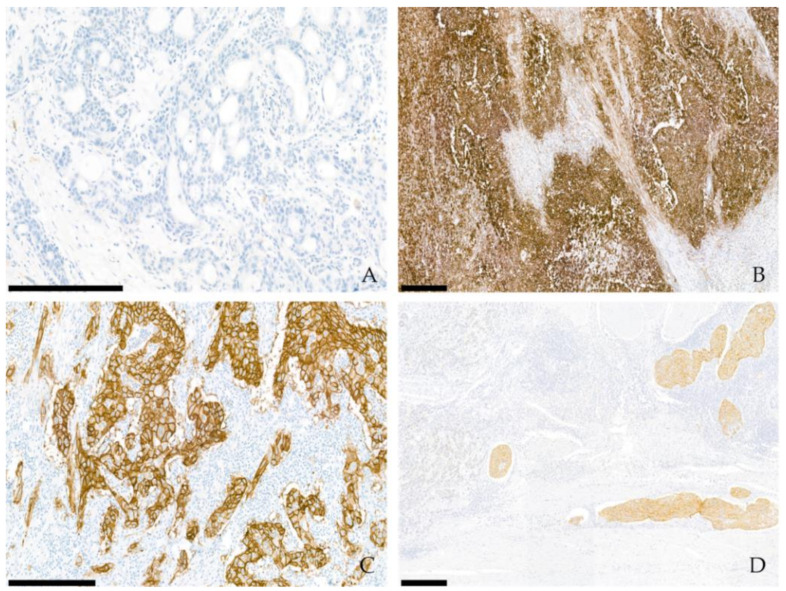
Representative microscopic findings of immunohistochemistry for PD-L1 (**A**) combined positive score 0; (**B**) combined positive score 90), for HER2 (**C**) with 3+ strong membranous staining in the tumor cells, and for FGFR2b (**D**) showing heterogeneous staining with positively stained tumor cells within lymphatic spaces. The scale length within the photograph is 300 µm.

Programmed death-1 (PD-1) is an inhibitory checkpoint receptor protein expressed on cytotoxic T cells and other immune cells [50]. Some tumor cells express high levels of PD-L1 as an immune evasion mechanism because PD-1/PD-L1 interaction induces the inactivation of cytotoxic T cells and the downregulation of immune responses [51]. The introduction of immune checkpoint inhibitors (ICIs) targeting PD-1 or PD-L1 has provided a paradigm shift in treatment strategies for several solid tumors, including GC [52]. PD-L1 protein expression is generally assessed by immunohistochemistry (IHC) for the selection of GC patients eligible for ICI treatment [9]. Thus, it is crucial that pathologists accurately interpret the PD-L1 assessment results. PD-L1 expression is evaluated by IHC in both tumor and immune cells [8]. For tumor cells, PD-L1 positivity is defined as a partial or complete membranous staining at any intensity. Tumor-associated immune cells (lymphocytes and macrophages) are considered to be PD-L1 positive when membranous and/or cytoplasmic staining is present at any intensity. The combined positive score (CPS), which is the number of PD-L1-stained cells (viable tumor cells, lymphocytes, and macrophages) divided by the total number of viable tumor cells multiplied by 100, is used to determine PD-L1 expression in GC (Figure 1A,B). Tumors are considered PD-L1 positive if the CPS one or higher. In G/GEJ cancer, 47.3–82.0% of patients showed PD-L1 positivity across studies [7,12,13,14]. Currently, four PD-L1 assays (22C3, 28-8, SP142, and SP263) are available for the detection of PD-L1 protein expression, and each assay predicts the clinical efficacy of four immunotherapy drugs (pembrolizumab, nivolumab, atezolizumab, and durvalumab, respectively) [10]. Although 22C3 PharmDx (Agilent, Santa Clara, CA, USA) is the only companion diagnostic method approved by the US Food and Drug Administration (FDA) for GC, recent studies have reported evidence of potential interchangeability of different PD-L1 assays [10,53,54].

As a biomarker, PD-L1 has some limitations that can affect its usefulness as a predictor of ICI response. One of these limitations is the intratumoral heterogeneity of PD-L1 expression. In GC, PD-L1 expression has been shown to display a high discordance rate not only between biopsy and surgically resected tissue but also between primary and metastatic tumors [55,56,57]. Temporal heterogeneity of PD-L1 expression between tumors before and after chemotherapy has also been reported [58]. For an accurate diagnosis of PD-L1 expression and an enhanced prediction of ICI responses, multiple biopsy samples (at least five) should be obtained in GC [55,56]. Another main limitation is the interobserver variation in PD-L1 expression evaluation. Several studies have reported a high discordance rate in PD-L1 expression estimation, especially around a clinically relevant cut-off of 1% [54,59,60,61]. These discrepancies occur even among expert pathologists. Thus, standardized and objective methods for PD-L1 assessment are urgently required. Recent studies have demonstrated that the application of artificial intelligence (AI)-based image analysis results in an excellent performance in scoring PD-L1 IHC and is comparable with assessments by pathologists [62]. Moreover, the AI model showed better predictions of tumor responses and survival outcomes of ICI in patients with non-small cell lung cancer (NSCLC) [63]. The development of AI and digital pathology can provide an accurate diagnosis with high repeatability and can be a standardized diagnostic method for PD-L1 interpretation.

Monoclonal antibodies against PD-1 (pembrolizumab and nivolumab) have demonstrated efficacy in patients with advanced GC in large clinical trials. The Phase II KEYNOTE-59 trial showed durable responses to pembrolizumab treatment in patients with advanced or metastatic PD-L1+ GC [12]. The tumors were considered to be PD-L1+ when the PD-L1 CPS with the 22C3 pharmDX assay was ≥1. The subsequent phase III KEYNOTE-61 trial found that pembrolizumab did not significantly improve the median overall survival (OS) compared to paclitaxel (9.1 vs. 8.3 months, hazard ratio (HR) 0.82, 95% confidence interval (CI) 0.66–1.03; one-sided *p* = 0.0421) for advanced GC with a PD-L1 CPS ≥ 1 [13]. Nonetheless, patients with a high PD-L1 CPS (≥10) had considerable therapeutic benefit compared to those with paclitaxel (median OS 10.4 vs. 8.0 months, HR 0.64, 95% CI 0.41–1.02), indicating that the proportion of tumor cells that express PD-L1 may affect the ICI treatment response [13]. In the phase III ATTRACTION-2 trial, nivolumab also resulted in meaningful improvements in the OS of previously treated advanced gastric and gastroesophageal junction (G/GEJ) cancer patients compared to a placebo as a third- or later-line treatment (median OS 5.3 vs. 4.1 months, HR 0.63, 95% CI 0.51–0.78; *p* < 0.0001) [11]. The study reported that a survival benefit was observed regardless of the degree of tumor PD-L1 expression. However, PD-L1 expression was assessed only in tumor cells (not in immune cells) in 40% of patients retrospectively, and the significance of PD-L1 expression and the nivolumab response was unclear. A recent phase III CheckMate 649 study evaluated the clinical outcome of nivolumab plus chemotherapy versus chemotherapy alone as a first-line treatment in patients with previously untreated, advanced, or metastatic G/GEJ cancer [14]. The study showed that the nivolumab plus chemotherapy arm had statistically significant improvements in both the median OS and progression-free survival (PFS) in patients with a PD-L1 CPS ≥ 5 (OS, HR 0.71, 98.4% CI 0.59–0.86; *p* < 0.0001, and PFS, HR 0.68, 98% CI 0.56–0.81; *p* < 0.0001). Additionally, patients with a PD-L1 CPS ≥ 1 showed a significant benefit for OS (HR 0.77, 99.3% CI 0.6–0.92; *p* = 0.0001). These results indicate that nivolumab also provides greater efficacy and benefits in patients with a higher PD-L1 CPS.

Another reliable predictive biomarker for anti-PD-1 ICIs in GC is the molecular subtype. Kim et al. investigated the determinants of treatment response to pembrolizumab in metastatic GC patients according to TCGA molecular subtypes [7]. Patients with EBV+ and MSI-H tumors displayed dramatic responses to pembrolizumab treatment (overall response rates [ORRs] of 100% and 85.7% in EBV+ and MSI-H GC, respectively) [7]. A subset of MSI-H patients with a heterogeneous microsatellite mismatch repair status failed to respond favorably to the treatment. The following study demonstrated the genomic, immunological, and clinical outcome heterogeneity within MSI-H GC and found that the number of whole-exome sequencing-derived nonsynonymous mutations, a diverse T cell receptor (TCR) repertoire, and increased PD-1+/CD8+ T cells correlate with antitumor activity and longer PFS with pembrolizumab [64]. Hence, comprehensive molecular and immunological characterization of GC, in combination with PD-L1 expression, is necessary to precisely and accurately predict ICI treatment.

TIL is also a key biomarker that can be assessed in conjunction with PD-L1. Previous studies have reported that high TIL density and increased numbers of CD3+ or CD8+ T cells are associated with favorable prognoses in GC patients [65,66]. Additionally, the distribution pattern of TILs is of interest in predicting the PD-L1 immunotherapy response in a variety of cancers. Several studies have suggested the concept of three immune phenotypes based on the status of the TIL distribution in tumors or peritumoral stroma: inflamed (TIL located intratumorally), excluded (TIL retained in the peritumoral stroma), and desert (sparse TIL in both tumor nests and stroma) [67,68,69]. Each phenotype displayed distinct biological characteristics, and tumors with an inflamed phenotype showed increased PD-L1 expression by tumor and immune cells as well as a better response to ICI treatment compared to other phenotypes [68,69]. Additionally, a recent TIL study of GC showed a significant association with the number of frameshift mutations, the tumor mutational burden, and OS [70]. Taken together, the amount of TIL, immune cell subtypes, and their distribution are important factors associated with prognostic significance and can be predictive markers of an immunotherapy response.

### 2.2. HER2

HER2 is a member of the human epidermal growth factor receptor family (HER1, HER2, HER3, and HER4) associated with tumor cell proliferation, adhesion, migration, and differentiation [71]. Hetero- or homodimerization of HER2 results in the autophosphorylation of the tyrosine kinase domain and subsequent activation of downstream signaling pathways [72,73].

Overexpression/amplification of HER2 has been identified in 7–53% of GC cases and varies between studies [74,75,76]. Overexpression of HER2 depends on the location (e.g., the upper third of the stomach), CIN subtype, differentiation (well or moderately differentiated), and histologic (intestinal) subtype [17,18]. Genomic alterations, such as *TP53*, *CDKN2A*, *PIK3CA*, and *KRAS* mutations, are associated with HER2+ GC [15,17]. Additionally, HER2+ GC has been reported to harbor the concurrent amplification of *CCNE1*, *PIK3CA*, *KRAS*, *CDK4*, and *CDK6* genes [15,17]. *TP53* mutation and *CCNE1* amplification were the most commonly identified single nucleotide variants and copy number alterations, respectively, in these studies. The alterations of *TP53* and *CCNE1* were more significant in HER2+ GC than in HER2- GC [76].

Currently, IHC and in situ hybridization (ISH) are recommended for the detection of HER2 overexpression/amplification [16]. HER2 immunostaining is generally assessed based on the score of the staining intensity (on a scale from 0 to 3+) and on the calculation of the proportion of stained tumor cells [77]. HER2 overexpression is defined as an IHC score of 3+ (Figure 1C). ISH should be performed to confirm the *HER2* gene amplification status in cases of equivocal HER2 staining (IHC 2+). ISH results are considered to be positive when the HER2/chromosome enumeration probe 17 (HER2/CEP17) ratio is greater than 2. If CEP17 polysomy (≥ 3 copies of CEP17) presents with HER2/CEP17 < 2, then HER2 ≥ 6 is considered to represent ISH positivity [16].

A major limitation of these slide-based HER2 assays is the intratumoral heterogeneity of HER2. Heterogenous HER2 overexpression is common in GC and has been reported to be present in 33–52% of cases [17,74,76]. The high incidence of HER2 heterogeneity is considered the main reason for the high discordance rate (12.3%) between paired biopsy and resection specimens. This discrepancy has also been identified in 20% of the paired primary and distant metastatic HER2+ GC [74]. For the accurate prediction of HER2 positivity in GC, multiple biopsy fragments (at least four) are needed, and HER2 IHC assays should be performed in both biopsy/resection specimens and in primary/metastatic sites [74,78]. Additionally, intratumoral heterogeneity of HER2 expression can induce false-negative results for *HER2* amplification by next-generation sequencing (NGS) [76]. As a low tumor heterogeneity index (the H-score of HER2 IHC multiplied by the tumor volume) affects the detection rate of HER2 amplification by NGS, an adequate tumor heterogeneity index is required [76].

Of the various biomarkers in use, HER2 overexpression/amplification is particularly important in GC because the targeted therapy trastuzumab is well-established for the treatment of HER2+ GC [19]. Trastuzumab is a monoclonal antibody that binds to the extracellular domain of the HER2 receptor and inhibits the HER2 signaling pathway. A multicenter phase III ToGA trial demonstrated significantly improved survival in HER2+ advanced G/GEJ adenocarcinoma patients who received traditional chemotherapy plus trastuzumab compared to patients who were treated with chemotherapy alone (median OS 13.8 vs. 11.1 months, HR 0.74, 95% CI 0.60–0.91; *p* = 0.0046) [19]. Based on this trial, trastuzumab is considered to be a standard first-line treatment option for patients with HER2+ GC. Recently, the antibody-drug conjugate trastuzumab-deruxtecan, consisting of an anti-HER2 antibody, a cleavable tetrapeptide-based linker, and a cytotoxic topoisomerase I inhibitor, demonstrated significant improvements in overall response and in OS in previously treated HER2+ GC patients [20]. The patients in the trastuzumab-deruxtecan treatment group had an ORR of 51% and a significantly longer median OS than patients in the chemotherapy-only group (12.5 vs. 8.4 months, HR 0.59, 95% CI 0.39–0.88; *p* = 0.01). The FDA approved trastuzumab-deruxtecan for HER2+ G/GEJ cancer based on these findings.

However, several HER2-targeting agents other than trastuzumab, including lapatinib, pertuzumab, and trastuzumab-emtansine, used as first- or second-line treatments failed to improve clinical outcomes in HER2+ GC patients [79,80]. The following underlying resistance mechanisms may have contributed to the disappointing results of HER2-targeted therapy and the aggressive behavior of tumors: (1) intratumoral heterogeneity of HER2, (2) aberrant activation of the *PIK3CA* signaling pathway (downstream signaling of HER2), and (3) concurrent amplification of *EGFR*, *MET*, and *CCNE1* [79]. Novel HER2-targeted therapies, including bispecific antibodies (ZW25), antibody-drug conjugates (RC48-ADC), and pan-HER tyrosine kinase inhibitors (TKIs) (afatinib, neratinib, and tucatinib), have been designed to overcome this resistance, and they have proven to be effective in clinical trials [79,80]. In future studies, the simultaneous use of targeted agents for co-existing mutations or amplifications in-line with anti-HER2 drugs can prove to be promising ways to overcome HER2 therapy resistance.

A recent phase III trial (KEYNOTE-811) reported that pembrolizumab could benefit patients with HER2+ GC [21]. In KEYNOTE-811, patients with previously untreated advanced or metastatic HER2+ G/GEJ cancer received trastuzumab/chemotherapy plus either pembrolizumab or a placebo. The ORR was 74.4% in patients who received pembrolizumab compared to 51.9% for those who received the placebo [21]. Additionally, the pembrolizumab-treated patients showed a marked reduction in tumor size and a higher complete response rate (11.3% for pembrolizumab vs. 3.1% for the placebo) [21]. The FDA granted accelerated approval to pembrolizumab for HER2+ GC based on this trial. The KEYNOTE-811 trial demonstrated that adding an ICI to targeted molecular therapy could be another efficient strategy to overcome HER2-resistance in GC [21].

### 2.3. VEGFR2

VEGFR2 is a receptor tyrosine kinase which regulates angiogenesis. Activation of the VEGFR2 signaling pathway contributes to new vessel formations to promote the transportation of nutrients, oxygen, and growth factors necessary for tumor survival, proliferation, and metastasis [81]. In GC, the positive rate of VEGFR2 expression by IHC in tumor cells are varied in studies (range: 0–53.5%) [24,82,83,84,85]. In contrast, VEGFR2 positivity in neoplastic endothelial cells was observed in approximately 50% of GC cases [24,83]. It remains controversial whether VEGFR2 expression in tumor cells or endothelial cells is an independent prognostic factor in GC patients, due to conflicting data across studies [24,82,83,84,85].

Ramucirumab is a human IgG1 monoclonal antibody against VEGFR2 which prevents ligand binding and activation signaling pathways in endothelial cells [86]. Two phase III trials (RAINBOW and REGARD) showed the efficacy of ramucirumab for advanced G/GEJ cancer patients [22,23]. In the REGARD trial, the ramucirumab monotherapy group exhibited a significantly improved median OS (5.2 vs. 3.8 months, HR 0.78, 95% CI 0.61–0.1; *p* = 0.047) in previously treated advanced G/GEJ adenocarcinoma [22]. Similarly, the RAINBOW trial demonstrated that ramucirumab in combination with paclitaxel as a second-line therapy significantly increased the median OS (9.6 vs. 7.4 months, HR 0.81, 95% CI 0.68–0.96; *p* = 0.017) compared to that of the placebo plus paclitaxel [23]. It is noteworthy that patients in the REGARD and RAINBOW trials were not selected based on the VEGFR2 expression status. Ramucirumab is currently used as a second-line treatment for biomarker-unselected G/GEJ cancer [2]. Apatinib, a TKI that selectively targets VEGFR2, showed an improved median OS (6.5 vs. 4.7 months, HR 0.71, 95% CI 0.54–0.94; *p* = 0.015) compared to that of the placebo in previously treated, biomarker-unselected patients with G/GEJ cancer in a randomized phase III trial from China [87]. However, a subsequent international phase III study (ANGEL) showed no survival benefits of Apatinib administration in advanced/metastatic GC patients [88]. Another TKI, Fruquintinib, which inhibits VEGFR1-3, demonstrated its efficacy in combination with paclitaxel as a second-line treatment in patients with advanced GC in a phase I/II study [25]. Fruquintinib is currently under phase III trial (NCT03223376). 

Fuchs et al. retrospectively analyzed the predictive biomarkers in REGARD G/GEJ carcinoma patients [24]. They investigated VEGFR2 IHC in tumor tissue samples as well as VEGF and soluble VEGFR levels in serum samples. However, none of the biomarkers showed a statistically significant association with ramucirumab efficacy. VEGFR2 protein expression in the tumor nuclei, cytoplasm, and membrane was minimal, and the number of positive samples was too small for correlative analysis with the patients’ survival. Ramucirumab treatment was associated with a trend toward longer survival in both high and low VEGFR2 endothelial expression subgroups. Similarly, Van Cutsem et al. performed a predictive biomarker analysis in patients with advanced GC from the RAINBOW trial [89]. This study evaluated various circulating factors, including VEGF in patients’ plasma samples, but did not find any biomarkers related to ramucirumab response. These findings support that both serum VEGF and VEGFR levels as well as both VEGFR2 expression in tumor and vessels cannot accurately predict the response of anti-VEGFR2 treatment in advanced GC patients. Thus, the proportion of patients who are eligible for the ramucirumab treatment cannot be estimated based on VEGFR2 expression. Recent studies suggest that molecular profiles and tumor immune microenvironments can be helpful for stratifying clinical responses to ramucirumab treatment. Tada et al. reported that GC patients who showed responses to ramucirumab and longer PFS had a higher population of regulatory T cells within TIL compared to non-responders [90]. Another study by Kim et al. suggested that EBV-positivity and somatic mutation of GNAQ is significantly associated with ramucirumab sensitivity for GC [91]. Future research in molecular and immunological characterization of GC can reveal more efficient predictive markers for antiangiogenic therapies.

### 2.4. MET

The *MET* proto-oncogene encodes a receptor tyrosine kinase protein called hepatocyte growth factor (HGF) [92]. MET activation triggers a downstream cascade of PI3K and RAS signaling and regulates cell survival and proliferation [93]. Thus, the over-activation of MET plays a critical role in cancer development and is frequently identified in various types of tumors, including GC [94]. MET overexpression and amplification are significant prognostic indicators of poor survival outcomes in GC patients [26,27,29].

In GC, MET protein overexpression, as determined by IHC, has been observed in up to 63% of cases [33], whereas the amplification of the *MET* gene is rare (~4%) [34], which reflects the possibility of discordance between protein overexpression and copy number gain. Many studies have evaluated MET protein overexpression by the assessment of the intensity of membranous immunostaining of tumor cells. Ha et al. found that MET protein overexpression based on membranous and cytoplasmic staining significantly correlated with high mRNA levels (r = 0.465, *p* < 0.0001), increased copy number gain (r = 0.393, *p* < 0.0001), and *MET* gene amplification in GC [26]. Application of the aforementioned interpretation method may reduce the discrepancy between MET overexpression and amplification. *MET* exon 14 skipping mutation is another mechanism that can result in discordance between protein overexpression and low gene amplification [28]. This mutation delays the ubiquitination and degradation of the MET protein, leading to its overexpression and aberrant activation. The frequency of *MET* exon 14 skipping mutations was investigated in a series of 230 solid tumor specimens, including 42 GC specimens. A total of 13 out of 230 tumors (5.7%) and 3 out of 42 GC (7.1%) had *MET* exon 14 skipping mutations. Notably, MET protein overexpression by IHC was identified in all *MET* exon 14-positive cases, whereas gene amplification was reported in only one case [28].

Clinical trials have used both MET protein expression level (by IHC) and *MET* gene amplification (by ISH, NGS, or circulating tumor DNA (ctDNA) examination) as criteria to select patients who were eligible for MET-targeted therapies. Several trials have evaluated the efficacy of monoclonal antibodies that target MET in patients with advanced or metastatic MET+ GC. Onartuzumab and rilotumumab, which target MET and MET-ligand HGF, respectively, have been used in phase III trials as first-line treatment in combination with conventional chemotherapy but failed to improve the clinical outcome of GC patients with MET overexpression [95,96]. In a phase II study, emibetuzumab, which blocks both ligand-dependent and ligand-independent MET signaling pathways, showed limited single-agent activity [97]. In these trials, the definition of MET positivity was confirmed by IHC but varied in intensity and proportion. 

Additionally, selective MET-TKIs have been studied in GC patients. A highly selective small-molecule MET inhibitor, AMG 337, showed 18% ORR in advanced G/GEJ cancer patients with *MET* gene amplification in a phase II trial [31]. Savolitinib, a small-molecule inhibitor of MET kinase, demonstrated promising antitumor activity in *MET*-amplified cancer cells and in GC patients in phase II VIKTORY umbrella trials [30]. In the VIKTORY study, GC patients with *MET* amplification had high response rates (10/20, ORR 50%; 95% CI 28.0–71.9) to savolitinib monotherapy, and 70% of the responders had an enhanced *MET* copy number (>10 copies) [30]. The study also noted that coexisting receptor tyrosine kinase amplification, other than *MET* amplification, resulted in a short response or no response to savolitinib [30]. Thus, the use of MET TKIs with other agents targeting concomitant genomic alterations should be considered in future studies. Another promising result of TKIs was obtained with crizotinib, which functions as a multi-kinase inhibitor and is usually used for the treatment of ALK- or ROS1-positive NSCLC [98,99]. A study of patients with *MET*-amplified NSCLC demonstrated that a high-level amplification group (MET/CEP7 ratio ≥ 4) responded to crizotinib with the highest ORR compared to medium- or low-level groups [100]. In GC, Aparicio et al. evaluated the efficacy of crizotinib in nine patients (six gastric and three esophageal adenocarcinoma) with *MET* amplification (≥6 copies), and the best ORR was 55.6% (95% CI 21.2–86.3), with a median PFS of 3.2 months (95% CI 1.0–5.4) and an OS of 8.1 months (95% CI 1.7–24.6) [32]. Additionally, alteration of *MET* exon 14 can be a potential candidate for targeted treatment. Two MET-TKIs, capmatinib and tepotinib, were approved in 2020 for the treatment of NSCLC harboring *MET* exon 14 skipping mutations [101]. In GC, patient-derived tumor cell lines exhibited profound inhibition of growth by MET inhibitors [28]. Although only a small subset (7.1%) of GC patients harbor *MET* exon 14 mutations, targeted agents for this unique alteration may provide clinicians and patients with new treatment strategies.

### 2.5. FGFR2 

FGFR2 is a member of the fibroblast growth factor receptor (FGFR) family of transmembrane tyrosine kinase receptors that regulate cell survival, differentiation, and proliferation [102,103]. Genomic aberrations of *FGFR2*, including amplification, point mutation, and oncogenic fusions, have been identified in various types of cancers, including GC [104]. Among the genomic alterations, *FGFR2* gene amplification is known to be a poor prognostic factor in GC patients, and a high copy number (≥30) is significantly associated with a shorter PFS and OS [39]. The prevalence of *FGFR2* amplification is rare in GC, which varied from 4 to 7% in previous studies, depending on the country [36,39].

Detection of *FGFR2* amplification is usually performed at the molecular level by ISH or NGS. These molecular-based analyses are time-consuming and expensive as screening methods. Considering the low frequency of *FGFR2* amplification and its poor concordance with *FGFR2* mRNA expression [105], IHC could be a faster and more efficient screening tool to stratify patients with GC who are likely to benefit from FGFR2-targeted therapy. Ahn et al. reported that FGFR2b IHC staining strongly correlates with *FGFR2* copy number alterations with high sensitivity and specificity [37]. Membranous staining and cytoplasmic staining were evaluated for positive FGFR2b expression. FGFR2b overexpression and a high H-score by IHC are also significantly associated with an advanced N stage and a shorter survival outcome, which is consistent with the results of *FGFR2* gene amplification [37]. Hence, recent clinical trials have mainly used FGFR2b overexpression by IHC as a patient eligibility criterion.

Intratumoral heterogeneity of FGFR2b protein expression is frequent and has been observed in 55.5% of GC cases [106]. Interestingly, an analysis of matched primary and metastatic GC cells revealed more FGFR2b+ tumors at metastatic sites than primary GC [37]. FGFR2b overexpression was also frequently found in lymphatic tumor emboli (Figure 1D) [37]. This finding suggests that *FGFR2* amplification may play a crucial role in tumor progression and lymphangitic metastasis in GC. Thus, tissue acquisition from distant metastases or primary tumors with extensive lymphovascular invasion could be adequate to accurately assay FGFR2 by IHC.

Preclinical studies have shown that AZD4547, a selective TKI of FGFR1, 2, and 3, has antitumor activity in *FGFR2*-amplified GC cell lines and in patient-derived xenograft models [107,108]. However, a randomized phase II SHINE trial that compared the outcome of AZD4547 versus paclitaxel as a second-line treatment for advanced G/GEJ cancer with *FGFR2* amplification or polysomy did not find that there was a survival benefit in the AZD4547 arm [105]. Recently, a novel FGFR2 inhibitor called bemarituzumab, which is an IgG2 monoclonal antibody that targets FGFR2b, showed promising results in GC patients with *FGFR2* gene amplification in a phase II clinical trial (FIGHT trial) [38]. In the FIGHT trial, patients with locally advanced or metastatic GC with FGFR2b overexpression (with any 2+ or 3+ staining detected by IHC) or *FGFR2* amplification (detected by circulating tumor DNA) were included and treated with bemarituzumab in combination with mFOLFOX6 as a first-line therapy, and the survival outcome was compared to the mFOLFOX6 plus placebo group. The median PFS was increased to 9.5 months in the bemarituzumab-treated patients compared to 7.4 months in the placebo group (HR 0.58, 95% CI 0.35–0.95; *p* = 0.03). The bemarituzumab arm also showed an improved median OS of 19.2 months vs. 13.5 months for the placebo group (HR 0.60, 95% CI 0.38–0.94). Interestingly, in a subset of patients with ≥10% FGFR2b overexpression by IHC, the median OS for the bemarituzumab arm was 25.4 months compared to 11.1 months for the placebo (HR 0.41, 95% CI 0.23–0.74) [40]. This finding indicates that the higher the proportion of tumor cells exhibiting FGFR2b expression, the greater the effect of targeted therapy. Additionally, the FIGHT trial mentioned that patients with FGFR2b overexpression benefited from bemarituzumab administration regardless of their ctDNA gene amplification status. Hence, the detection of gene amplification by ISH, NGS, or ctDNA assays may diminish in importance in future studies. 

### 2.6. CLDN18.2

Claudin family proteins are one of the main components of tight junction complexes [109]. Aberrant tissue expression of claudin proteins can lead to impaired tight junction functions, can affect cell signaling pathways, and can contribute to neoplastic progression in some epithelial cancers [110,111,112,113,114]. CLDN18.2 is a member of the claudin family and is expressed exclusively in tight junctions of gastric mucosa [113]. GC and pancreatic cancer cells commonly express CLDN18.2 [115]. Activation of the protein kinase C/mitogen-activated protein kinase signaling pathway is associated with upregulation of CLDN18.2 in GC cells [116]. However, the definite biological role of CLDN18.2 expressed on the surface of GC cells and how CLDN18.2 expression contributes to tumor progression are still unclear. Nevertheless, one of the well-studied genomic alterations, *CLDN18-ARHGAP* fusion, is known to be associated with CLDN18.2 expression in GC [117]. The chimeric protein CLDN18-ARHGAP is not able to interact with actin regulatory proteins from the tight junction complexes, and this results in the loss of cell-cell adhesion and in the gain of an epithelial-mesenchymal phenotype [117,118,119]. Inhibition of the RHOA signaling pathway by Rho-GAP of ARHGAP is also known to contribute to carcinogenesis [117,118,119]. *CLDN18-ARHGAP26/6* fusion has been detected in up to 15% of G/GEJ cancer patients, predominantly in the GS subtype and among young patients with poor prognoses [4,117]. CLDN18.2 expression has been reported in almost all *CLDN18-ARHGAP* fusion-positive GCs [117,120]. In GC, high CLDN18.2 expression has been reported to be present in 14.1–51.5% of cases and varies across studies [41,42,43,44,45,121,122]. CLDN18.2 expression was significantly associated with a diffuse histologic type, non-antral location, and EBV-positivity [41,42,43]. A majority of the studies reported that high expression of CLDN 18.2 was not associated with patient survival [42,121,122,123,124].

Zolbetuximab is a chimeric IgG1 monoclonal antibody that binds to CLDN18.2 molecules on the tumor cell surface and induces antibody-dependent cellular cytotoxicity and complement-dependent cytotoxicity [44,45]. Two recent phase II clinical trials have demonstrated the efficacy and safety of zolbetuximab. In the FAST trial, zolbetuximab was used with epirubicin, oxaliplatin, and capecitabine in advanced G/GEJ and esophageal adenocarcinoma patients, significantly prolonging the PFS (7.5 vs. 5.3 months, HR 0.44, 95% CI 0.29–0.67; *p* < 0.0005) and OS (13.0 vs. 8.3 months, HR 0.55, 95% CI 0.39–0.77; *p* < 0.0005) of the patients compared to chemotherapy alone [45]. The MONO trial, in which zolbetuximab single therapy was used, documented that 9% of locally advanced metastatic GC patients with CLDN18.2 expression achieved clinical benefits from the treatment [44]. Based on these encouraging results, two phase III trials are in progress to evaluate the efficacy of zolbetuximab plus chemotherapy as a first-line treatment in patients with CLDN18.2+/HER2-, locally advanced, unresectable, or metastatic G/GEJ adenocarcinoma [125,126].

In previous studies, CLDN18.2 expression in tumor cells was only determined by IHC [124]. Membranous staining of moderate to strong (2+/3+) intensity was regarded as high expression in tumor cells, and the proportion of CLDN18.2+ cells was calculated. Although the clinically relevant cut-off value for CLDN18.2+ GC has not yet been established and varies between studies, a cut-off value of approximately 70% can be recommended based on the FAST and MONO trial results [44,45]. In the FAST trial, which enrolled patients with CLDN18.2 expression in ≥40% of tumor cells, the magnitude of the survival benefit was better in patients with CLDN18.2 expression in ≥70% of the tumor cells (median OS 16.5 vs. 8.9 months, HR 0.50, 95% CI 0.33–0.74; *p* < 0.0005) than in the overall population [45]. Similarly, in the MONO trial, patients with high CLDN18.2 expression (≥70%) showed higher ORR (14%), and in all the best responders ≥70% of tumor cells had CLDN18.2 immunoreactivity [44]. The ongoing phase III trials are using a cut-off value of ≥75% for CLDN18.2+ patients [125,126]. The optimal cut-off values are expected to be established through these trials and future studies.

Aberrant nuclear or cytoplasmic CLDN18.2 expression in IHC, as well as heterogeneity, is frequent in GC [41,42]. Strong nuclear or cytoplasmic CLDN18.2 positivity was detected in 22.5% of cases, and intratumoral variability of membranous CLDN18.2 expression was found in 33.6% of cases [41]. Additionally, Dottermusch et al. noted that the intensity of CLDN18.2 immunostaining decreased towards the invasive front of the tumor in many cases, which may cause diagnostic problems for biopsy examinations [42]. Although the clinical significance of abnormal expression and intratumoral heterogeneity of CLDN18.2 is unclear, these features can represent diagnostic pitfalls for interpretation and challenges for selecting patients eligible for treatment.

### 2.7. TIL and Adoptive Cell Therapy

The significance of TIL as a predictive biomarker for immunotherapy was briefly discussed in the PD-L1 section. An increased number of TILs is frequently identified in EBV+ and MSI-H GC and is associated with a favorable response to ICI treatment and prolonged OS. A recent study investigated the distinct immunological profiles of GC using TCGA data [3]. EBV+ and MSI-H tumors displayed intense T cell infiltration, whereas CIN tumors contained few CD8+ T cells but abundant CD68+ macrophages. The GS group displayed enrichment of CD4+ T cells, tumor-associated macrophages, and B cells, and approximately 50% of the cases had tertiary lymphoid structures. Current anti-PD1 treatments with pembrolizumab showed less of an effect in CIN or GS subtypes than in EBV or MSI subtypes [7]. Thus, more effective immunotherapy is needed for anti-PD-1-resistant metastatic GC patients with CIN or GS subtypes.

A novel potent strategy, called TIL therapy, is based on adoptive cell therapy using TILs [46,49]. For this TIL therapy, TILs were isolated from the resected tumor specimen, expanded in culture with interleukin-2 to a clinically relevant level, and then infused back into the patients [46,49]. The advantages of TIL therapy for treating solid tumors are as follows: (1) TILs express diverse TCR repertoires that are capable of recognizing multiple tumor antigens, thereby overcoming the high intratumoral heterogeneity that tends to result in resistance to targeted therapy. (2) TILs have been stimulated by tumor antigens in vivo and are predominantly composed of effector memory T cells, which have chemokine receptors on the cell surface, thereby resulting in better tumor-homing ability. (3) There have been few reports to-date regarding TIL therapy off-target toxicity, and the negative selection of TCR of TIL may contribute to the safety of this treatment [49].

Several trials have reported the clinical benefits of TIL therapy, mainly in metastatic melanoma [47,48]. A recent phase II study demonstrated that Lifileucel (LN-144), an autologous TIL product, showed durable responses in patients with unresectable or metastatic melanoma who had been previously treated with ICIs [48]. TIL treatment has also been reported to be effective with other solid tumors, including cervical cancer, NSCLC, colorectal cancer, and breast cancer [127,128,129,130]. Currently, four clinical trials are ongoing for GC patients [49]. The application of novel TIL treatments can address an unmet need in advanced GC patients who are refractory to anti-PD-1 or other targeted therapies.

## 3. Conclusions

We reviewed the established and emerging biomarkers for GC treatment. GC is a histologically, molecularly, and immunologically heterogeneous disease. Various targeted therapies and biomarkers have been discovered based on an in-depth understanding of tumor molecular biology. Recent studies of the tumor immune microenvironment have also contributed to accurate predictions of chemotherapy and immunotherapy responses in GC. Targeted therapies for PD-L1 and HER2 have shown significant survival benefits in advanced GC patients with well-established diagnostic criteria. Anti-angiogenic therapy has demonstrated its efficacy in unselected patients, but additional predictive markers for treatment response should be further explored. Therapeutic agents targeting emerging biomarkers have remarkably improved patient survival or have achieved high ORR in clinical trials, but standardized diagnostic methods and interpretation guidelines should be established. Understanding the mechanisms of biomarkers, current diagnostic methods with limitations, and their implications for targeted therapy response is crucial in precision medicine. Comprehensive examination of these biomarkers in GC can provide better patient stratification and selection, allowing them to benefit from specific targeted therapies.

## Figures and Tables

**Table 1:** Representative features of the four gastric cancer subtypes based on The Cancer Genome Atlas (TCGA).

TCGA Subtype	EBV	MSI	CIN	GS	Ref.
Relative frequency	~9%	~22%	~50%	~20%	[4]
Typical histological features	GC with lymphoid stroma	None	Intestinal type	Diffuse type	[4]
Frequent location	Fundus and body	Distal location	GEJ/cardia	Distal location	[4]
Clinical characteristics	Best prognosis Potential benefit from ICI treatment	Favorable prognosis Lack of benefit from chemotherapy Highest benefit from ICI treatment	Greatest benefit from adjuvant chemotherapy	Worst prognosis Least benefit from adjuvant chemotherapy	[5,7]
Representative molecular alterations and methylation	CIMP *CDKN2A* silencing Frequent *PIK3CA* (~80%), *ARID1A* (~55%), and *BCOR* (~23%) mutation	CIMP MLH1 silencing Frequent genomic mutations/alterations	High *TP53* mutation (~71%) RTK amplifications (*EGFR*, *ERBB2*, *ERBB3*, *FGFR2*, *MET*, and *JAK2*)Amplification of cell cycle genes, *KRAS*/*NRAS*, and *VEGFA*	Recurrent *CDH1* (~37%) and *RHOA* (~15%) mutation*CLDN18-ARHGAP26/6* fusion (~14%)	[4]
Relevant tumor immune microenvironment	Increased TILs with intense T cell infiltrates	Increased TILs with intense T cell infiltrates	Increased tumor associated macrophages CD8+ T cells predominantly at the invasive margin	Enrichment of CD4+ T cell, macrophages and B cells Tertiary lymphoid structures (~50%)	[3]

Abbreviations: EBV: Epstein—Barr virus; MSI: microsatellite instable; CIN: chromosomal instability; GS: genomically stable; GC: gastric cancer; GEJ: gastroesophageal junction; ICI: immune checkpoint inhibitor; CIMP: CpG island methylator phenotype; RTK: receptor tyrosine kinase; TIL: tumor-infiltrating lymphocytes.

**Table 2:** Key biomarkers in gastric cancer with their clinicopathological relevance, alterations, diagnosis, and targeted agents.

Biomarker	Clinicopathological Relevance	Activation Mechanism (Frequency, %)	Detection Methods	Representative Therapeutic Agents (Survival Benefit in Months *)	Ref.
**Established Biomarkers**					
PD-L1	EBV and MSI subtypeIncreased TILs (Inflamed phenotype)	Overexpression (47–82%)	IHC (membranous staining for tumor, membranous and/or cytoplasmic for immune cells)	Pembrolizumab (CPS ≥ 1; 0.8 months, CPS ≥ 10; 2.4 months) Nivolumab (all pts; 1.8 months, CPS ≥ 1; 2.7 months, CPS ≥ 5; 3.3 months)	[7,8,9,10,11,12,13,14]
HER2	Upper third of the stomach, CIN subtype, Intestinal type	Overexpression/Amplification (7–53%)	IHC (membranous staining) FISH, NGS	Trastuzumab (all pts; 2.7 months, FISH +/IHC 2+ or IHC3+; 4.2 months) Trastuzumab deruxtecan (4.1 months)Trastuzumab + Pembrolizumab (NA)	[15,16,17,18,19,20,21]
VEGFR2	NA	Overexpression ^†^ (tumor cell; 0–54%, endothelial cell; ~50%)	IHC ^†^ (nuclear, cytoplasmic, or membranous staining for tumor, cytoplasmic for endothelial cells)	Ramucirumab (2.2 months)	[22,23,24,25]
**Emerging Biomarkers**					
MET	CIN subtype, Intestinal type Prognostic indicators of poor survival co-amplification in *EGFR*, *HER2*, and other RTK	Overexpression (22–63%) Amplification (2–3%) Exon 14 skipping Mutation (~7%)	IHC (membranous and/or cytoplasmic)FISH, NGS, ctDNA	AMG 337 (NA)Savolitinib (NA)Crizotinib (NA)	[26,27,28,29,30,31,32,33,34,35]
FGFR2	GS (9%) > CIN (8%) Diffuse type > intestinal type Predictor for poor prognosisAssociated with lymphatic metastasis	Overexpression (FGFR2b; 4%) Amplification (4–7%)	IHC (membranous)FISH, NGS, ctDNA	Bemarituzumab (all pts; 5.7 months, FGFR2b ≥ 10%; 14.3 months)	[36,37,38,39,40]
CLDN18.2	GS subtype, Diffuse type, non-antral location, EBV positivityNo correlation with survival outcome	Overexpression (14–52%)	IHC (membranous)	Zolbetuximab (all pts; 4.7 months, CLDN18.2 ≥ 70%; 7.6 months)	[41,42,43,44,45]
TIL	EBV and MSI subtype	-	-	TIL therapy (NA)	[3,46,47,48,49]

Abbreviations: EBV: Epstein—Barr virus; MSI: microsatellite instable; TIL: tumor-infiltrating lymphocyte; IHC: immunohistochemistry; CPS: combined positive score; CIN: chromosomal instability; FISH: fluorescence in situ hybridization; NGS: next-generation sequencing; NA: not applicable; RTK: receptor tyrosine kinase; ctDNA: circulating tumor DNA; GS: genomically stable. * Best survival benefit in months (prolonged median overall survival in targeted therapy group compared to control group) reported from the key clinical trials. ^†^ Not predictive for representative targeted drugs.

## Data Availability

Not applicable.
